# Carrageenan-Based Compounds as Wound Healing Materials

**DOI:** 10.3390/ijms23169117

**Published:** 2022-08-14

**Authors:** Bogdan Neamtu, Andreea Barbu, Mihai Octavian Negrea, Cristian Ștefan Berghea-Neamțu, Dragoș Popescu, Marius Zăhan, Vioara Mireșan

**Affiliations:** 1 Pediatric Research Department, Pediatric Hospital Sibiu, 550166 Sibiu, Romania; 2 Faculty of Medicine, “Lucian Blaga” University of Sibiu, 550169 Sibiu, Romania; 3 Faculty of Engineering, “Lucian Blaga” University of Sibiu, 550025 Sibiu, Romania; 4 Faculty of Animal Science and Biotechnologies, University of Agricultural Sciences and Veterinary Medicine Cluj-Napoca, 400372 Cluj-Napoca, Romania; 5 Department of Pediatric Surgery, Pediatric Hospital Sibiu, 550166 Sibiu, Romania; 6 Obstetrics and Gynecology Clinic, County Clinical Emergency Hospital, 550245 Sibiu, Romania

**Keywords:** carrageenan, heteroglycan, wound healing, biomaterial, infection control, nanofibers, pharmacokinetic

## Abstract

The following review is focused on carrageenan, a heteroglycan-based substance that is a very significant wound healing biomaterial. Every biomaterial has advantages and weaknesses of its own, but these drawbacks are typically outweighed by combining the material in various ways with other substances. Carrageenans’ key benefits include their water solubility, which enables them to keep the wound and periwound damp and absorb the wound exudate. They have low cytotoxicity, antimicrobial and antioxidant qualities, do not stick to the wound bed, and hence do not cause pain when removed from the wounded region. When combined with other materials, they can aid in hemostasis. This review emphasizes the advantages of using carrageenan for wound healing, including the use of several mixes that improve its properties.

## 1. Introduction

A biomaterial is considered to be “a natural or synthetic substance that is not a drug, which can be introduced into body tissue as part of an implanted medical device or used to replace, treat, or augment a tissue, an organ or a bodily function” [1].

Biomaterial-based wound dressings are able to hold drugs and antibiotics because they change their properties and release kinetics. Biomaterials used in wound healing should also assist the autolytic debridement while moisturizing the dry wounds and/or absorbing liquids and exudates from the wet ones. Moreover, if they promote angiogenesis in poor perfusion wounds, modulate the immune cells inside the wound, and enhance the fibroblasts and keratinocytes’ migration and invasion, thus preventing or treating a possible infection, then the healing process would be faster and smoother [2].

The ideal wound dressing has to be biocompatible, both bio-adherent to the wound surface and non-adhesive to the wound bed, making the removal painless and trauma-free, easy to apply and change, biodegradable, cost-effective, elastic but resistant, semi-permeable (allows gas exchange but stops micro-organisms), and compatible with therapeutic agents. It should also provide pain relief and a moist wound environment, protection against mechanical, bacterial, infectious, and thermal factors; moreover, it should exhibit debridement activity, non-antigenic and non-toxic properties, high excess exudate absorption, and healing/re-epithelialization capability. One of the most used materials that sum up most of these benefits are biopolymers because they are biodegradable and bioactive, and they promote tissue regeneration and wound healing through cell migration and proliferation [1,2,3,4,5,6,7,8]. “Smart” biomaterials can be used in 4D bioprinting, drug delivery, regenerative medicine, soft electronics, and even for artificial lives and synthetic biology [9,10,11].

Biopolymers, polymers produced by living organisms, are used either as standalone or in various combinations with other ingredients to maximize the benefits and lower the associated risks. The main disadvantage of natural polymers that are used for grafting blood vessels, suturing, or as a tissue substitute [12] is the fast deterioration rate; however, crosslinking them overcomes this characteristic. Crosslinking happens when two polymer chains are attached with chemical agents, resulting in a stronger bond. Because most of these biomaterials are obtained from animal sources, another concern is the probability of undesired side effects. Protein-based biomaterials usually have animal and human origins and are represented by bioactive molecules that resemble the extracellular environment, while polysaccharide-based biomaterials derive from algae (such as alginates, carrageenans, fucoidans, etc.), or microbial sources (dextran and its derivates) [13]. These polymers are modified for therapy in various degradable nontoxic forms such as hydrogels, particles, porous scaffolds, or thin membranes. The degradation kinetics are not easy to foretell, but the topical effects are remarkable [13].

**Figure 1:**
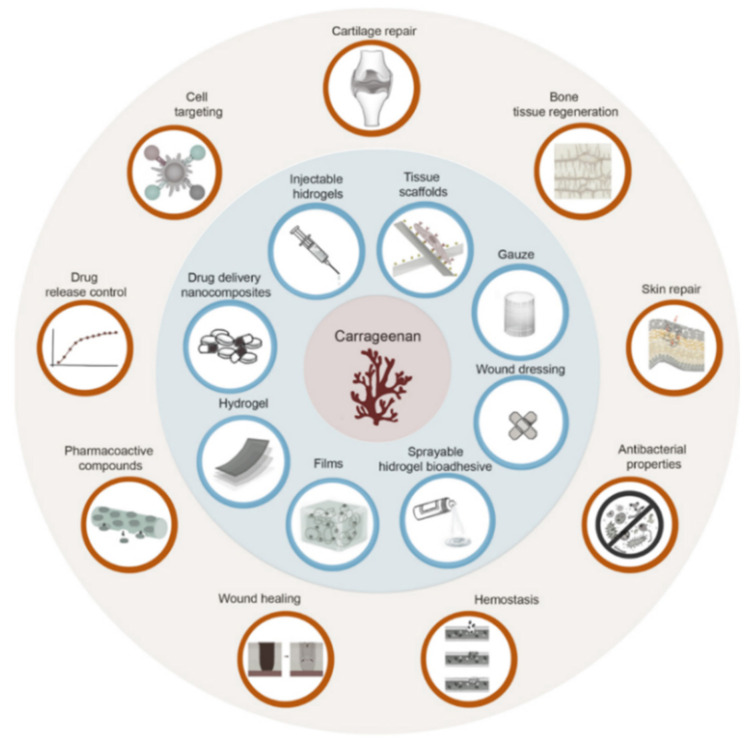
Applications (blue background) and biological uses or properties (red background) of carrageenan-based formulations.

Polysaccharide-based biomaterials used for wound healing can either be classified as homoglycans and heteroglycans or based on their pH [1,14]. Heteroglycans are high molecular weight polymers containing more than one type of monosaccharide residue. They are usually found in the connective tissue, cartilage, plant gums, cell walls, or some algae. Some heteroglycans are biocompatible, biodegradable, have specific physico-chemical characteristics such as thickening and gel- or film-assembling properties, and may form electrostatic networks, which makes them useful in biomedical or therapeutic applications. The most commonly used heteroglycans for therapy are alginates, carrageenans, fucoidans, glycosaminoglycans, gums, and pectins [1,15]. Figure 1 shows examples of various uses of carrageenans in biomedical applications.

## 2. Wound Healing

The current literature defines wounds as a damaging process involving the integrity of skin and mucous membranes and other deeper tissues. Researchers either describe the wound healing process as being comprised of either four overlapping phases—hemostasis, inflammation, proliferation, and remodeling—or three overlapping phases: (a) inflammatory, fibroblastic, and maturing, or (b) inflammatory, proliferation, and remodeling [16,17,18,19,20]. Wound contraction is the last stage of the wound healing process, usually overlapped with the remodeling phase [21,22].

**Figure 2:**
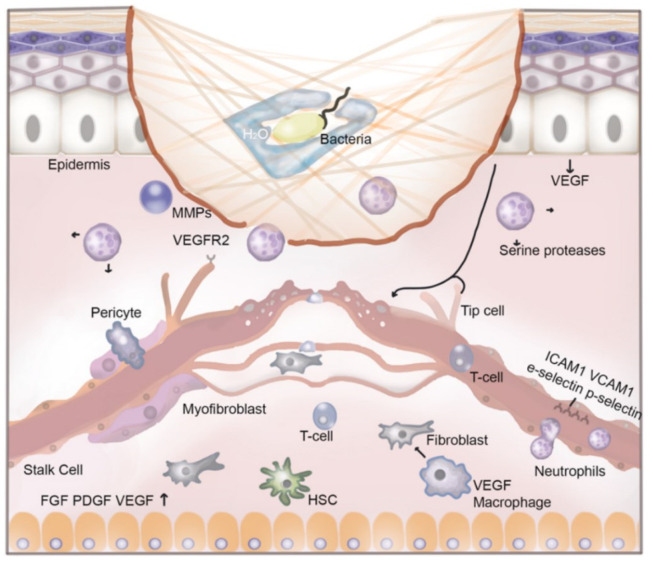
The process of wound healing.

Hemostasis, as depicted in Figure 2, happens when blood clots are formed on the wound, blood vessels contract, platelets aggregate, and a thrombus appears after degranulation and formation of fibrin [23]. In this phase, both the aggregation and degranulation of platelets and the release of alpha granules is increased in the elderly compared to the young patients. Alpha granules are platelets that include insulin-like growth factor 1 (IGF-1), platelet-derived growth factors, TGF-β, platelet factor 4 (a chemokine that binds heparin), and other coagulation proteins such as thrombospondin, fibronectin, and factor V [24,25].

Acute hypoxia right after an injury will lead to a momentary increase in cellular replication, but prolonging it will have detrimental effects in regard to wound healing. Hypoxia in fresh wounds enhances the production of cytokines and growth factors from fibroblasts, keratinocytes, and macrophages [21,26]. Cytokines produced as a result of hypoxia include endothelin-1, TNF-α (tumor necrosis factor-α), TGF-β, PDGF, and VEGF, all being crucial promoters of cell proliferation, migration, chemotaxis, and angiogenesis [27,28].

Inflammation, the first stage of wound healing after hemostasis, has a decontaminating role. If this contamination persists, the state of inflammation will last longer. *Staphylococcus aureus* (*S. aureus*) and *Pseudomonas aeruginosa* (*P. aeruginosa*) are the most common bacteria in infected wounds [21], while in the uninfected ones, β-hemolytic streptococci are present. Bjarnsholt et al. [29] consider that *P. aeruginosa* is responsible for the appearance of chronic ulcers because it blocks the phagocytic action of polymorphonuclear neutrophils (PMNs).

Figure 2 depicts the illustration of new blood vessel formation by endothelial cells responding to vascular endothelial growth factor (VEGF) and other intercellular signals from surrounding tissues. During angiogenesis, immune cells extravasate from the lumen into the wound. The following surface markers—intercellular adhesion molecule (ICAM)-1, vascular cell adhesion molecule (VCAM)-1, E-selectin, and P-selectin—play a crucial role in intercellular interactions leading to wound repair. Macrophages guide the process of angiogenesis by releasing growth factors and promoting the fusion between neo-vessels. Neutrophils respond to several signals including damage-associated molecular patterns, calcium waves, lipid mediators, hydrogen peroxide, and chemokines released by adjacent cells to the site of injury. They play an important antibacterial role by the release of proteases from intracellular granules, the production of neutrophil extracellular traps, and performing phagocytosis. Once their role has been achieved, neutrophil clearance is essential for the reduction of the inflammatory process. They can be destroyed by macrophages or they can return to circulation by reverse migration [19]. This figure also shows a representation of a carrageenan nanofiber-based wound dressing application within the lesion and illustrates the phenomenon of bacterial trapping. This incapacitates bacteria by immobilization in the network of nanofibers which swell after water absorption.

Wound healing mechanisms are also related to the dynamics in the bacterial biofilm found on the surface of the skin. Inflammation is a result of the inflammatory response of keratinocytes. Inflammation is also maintained by neutrophils, lymphocytes, and monocytes that infiltrate the wound bed and secrete biomarkers such as TNF-α, IL-1 (both pro-inflammatory cytokines), and IL-6 [28]. Growth factors and cytokines influence the migration, proliferation, and local differentiation of cells in this phase [30], while monocytes turn into essential macrophages [31].

Proliferation is the most critical stage, since collagen synthesizing, reepithelization, and angiogenesis begin, and the extra-cellular matrix (ECM) is formed [32]. If IL-1 or TNF-α are produced for an extended time, the wound may not heal and may become chronic. At the same time, the level of matrix metalloproteinases (MMPs) increases, and this can harm the extracellular matrix (ECM); with the increase in local proteinases, the number of protease inhibitors decreases [28,33].

Remodeling takes place when collagen reshapes, and the blood vessels mature and start to regress from the injured site, with reepithelization taking place [28,34]. Reepithelization is described by Hackam and Ford [35] as the regeneration of the continuity of the epithelium at the wound site when a waterproof seal is present on the cut surface of the wound. Angiogenesis, epithelization, and production of new glycosaminoglycans (GAG) and proteoglycans are vital to the site of wound healing. These processes result in the replacement of normal skin structures with fibroblastic-mediated scar tissue [36].

## 3. Carrageenans: Structure, Types, and Wound Healing Effects

**Figure 3:**
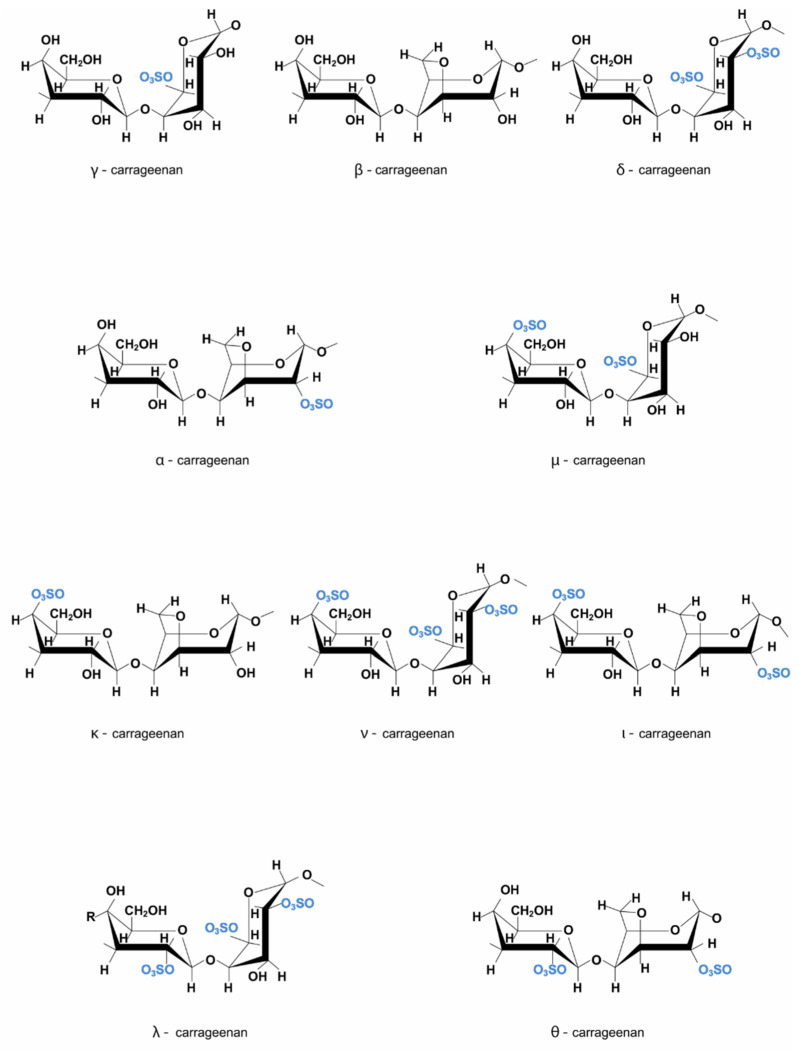
Carrageenan structure.

Carrageenans (CGs) are high molecular weight linear sulfated polysaccharides or marine hydrocolloids extracted from several red seaweed species, mostly from members of the *Rhodophyceae* class, such as *Chondrus crispus*, *Eucheuma cottonii*, *Eucheuma spinosum*, and *Gigartina stellate*, having anhydrogalactose and galactose units linked by glycosidic bonds [1,37,38,39,40]. CGs are hydrophilic D-sulfated galactans, polymers made of alternating 3-linked B-D-galactopyranose (G-units) and 4-linked a-D-galactopyranose (D-units) or 4-linked 3,6-anhydro-a-D-galactopyranose (DA- or AnGal-units), that create the disaccharide repeating unit of CGs. There are at least 15 types of CG, which differ in chemical structure, sulfation pattern, sulfate group number and place, presence/absence of AnGal-units, and seaweed source, such as iota (ι)-, kappa (κ)-, lambda (λ)-, mu (μ)-, nu (ν)-, and theta (θ)-carrageenan [39,41,42]. Iota-carrageenans [43] are sulfated at carbon-2, and ambda-carrageenans are highly sulfated and lack 3,6-anhydro galactose [37]. In addition to containing sulfate and galactose, carrageenans may also have glucose, methyl esters, pyruvate groups, uronic acids, and xylose residues. The molecular weight of commercially available CG varies between 100–1000 kDa [39]. Figure 3 illustrates the structure of the various carrageenan types.

Carrageenans [44], as well as alginates and pectins, have a type-A helix secondary structure, with a ribbon-like aspect, where polysaccharides link at β-(1,4), and they display water exclusion properties and have fairly strong inter-chain hydrogen bonds [45]. K- and I-CG assemble a network of 3D double helices because the sulfate groups are facing the exterior of the spiral chains and crosslinks [46,47], but the L-CG has the 2-sulfate group oriented towards the interior, and hence it avoids the crosslinking [39]. The general characteristics of carrageenans are summarized in Table 1.

Carrageenans, chitosan, and pectin fibers can form gel matrices absorbing water and solutes. The branching and spatial placement of the crosslinking blocks, concentration, ions, molecular weight, monosaccharidic content, temperature, and pH affect this gel network formation [45,58].

All CG fractions are water-soluble and insoluble in oil, organic solvents, or fats. CG water solubility is related to the sulfate group levels (very hydrophilic) and their associated cations. The solubility, gel strength, and solutions’ viscosity are influenced by the type of predominant ionizable cations found in CG and the balance between them—calcium (Ca^2+^), potassium (K^+^), sodium (Na^+^), and magnesium (Mg^2+^), as well as the sulfate fractions ratio, all of the above being relevant in the pharmaceutical and food industries usage of CG because of their rheological properties that make them act as a stabilizer, and a gelation and thickening agent [39,42,57]. The pharmacological or medical uses of CG for wound healing or tissue engineering include drug or growth factor delivery systems, enzyme confinement, and cell encapsulation for in vivo delivery [1]. By easily linking L- and K-CG with milk proteins, an enhanced solubility and better texture can be observed [56].

All of the lambda-CGs salt forms are water-soluble, regardless of the temperature [54]. The free acid is unstable, but the CG salt form has a more stable behavior, and Na^+^ forms of CG dissolve easier than those rich in K^+^. For example, the K salt of iota- and kappa-CG is not soluble in cold water and needs a temperature increase in order to transform the polysaccharide into a solution, but the Na form will dissolve instantly in the same condition. Kappa-CG [53] dissolves in hot water and will precipitate if K ions are added to the aqueous solution. Other solutes in the media will also influence the solubility and the dissolving rate of CG because they all contend for the same amount of solvent; hence, the polysaccharide’s hydration level will be affected [56].

A concentration increase will lead to a near logarithmic viscosity increase, ranging between 5 and 800 cps at 75 °C in 1.5% solutions and not less than 5 cps in the same conditions for CG used in the food industry [56]. The viscosity is also influenced by temperature, other solutes, CG type, and molecular mass, and this is a common behavior after charge or polyelectrolytes influencing the linear polymers. The mechanisms that influence the viscosity increase are: (A) linear chain interactions that lead to an increased excluded volume or decreased free space. The macromolecule concentration increase allows a strong interaction between chains, and the viscosity decreases with the presence of salts because the electrostatic repulsion among the sulfate groups is reduced—this mechanism is applied for λ fractions. (B) gelation as a consequence of inter-chain cross-linkage. Κ-, I-CG, and hybrid κ-2 fractions solutions can form higher viscosity gels at low temperatures and low concentrations of salt. Temperature influences the viscosity of a CG solution in an inversely proportional manner [39].

K-CG and I-CG can swell, form gels, and have a viscoelastic behavior when compressed. K-CG makes stronger, more rigid, and brittle gels in the presence of a few potassium (K) ions; I-CG forms elastic gels that, in calcium ions’ presence, will display a thixotropic behavior; L-CG does not form gels, but L-CG and I-CG can assemble right-handed helices. K-CG gels easier if KCl is present, and I-CG gels easier if Ca^2+^ ions are in the solution. An increased drug release rate was observed for diclofenac sodium inside a CG compacted tablet with high tensile strength and robustness if the viscosity increased and water retention and swelling capacity decreased [37,56]. The gelation temperature of K-CG ranges from 35–65 °C [45]. Adding locust bean gum will make the gel stronger, but other hydrocolloids can also affect gel tenacity and strength [56]. All obtained gels are thermally reversible; they begin formation after cooling to about 50 °C, and melt when they are heated at around 80–90 °C [46].

Until 1990, many pharmacokinetic articles regarding the oral intake of CG were conducted on rats, rabbits, guinea pigs, and Rhesus monkeys. Whether the tested rats were given native I-CG extracted from *E. spinosum* or degraded CG, the fecal excretion was similar, and untreated native K/L-CG extracted from *C. crispus* was also excreted through the feces after 10 days of administration. If the diet contained K/L-CG for either a month or 13 weeks, the researchers did not find any CG in the rats’ livers. After I-CG oral intake, no traces were detected in the small or large intestines of rats [56]. When guinea pigs were fed I-CG extracted from *E. spinosum* for 21–45 days, the CG was found in the macrophages located in cecal or colonic tissue (36–400 pg/g). When K-CG extracted from *C. crispus*, L-CG extracted from *C. crispus*, and I-CG extracted from *E. spinosum* were administered as a solution for 2 weeks, there were no traces found in the caecum; however, when a CG was given orally to new-born rabbits, traces of it were found in their liver, stomach, and small intestine, but no amounts were found in their cardiac or portal blood after 4 h from the treatment [56]. The Rhesus monkeys that were submitted to native CG (K/L-CG extracted from *C. crispus*) treatment in the drinking water for 7–11 weeks, followed by an 11-week recovery period, displayed no CG tissue storage. The same result was found when the K/L-CG treatment was given for 10 weeks with no recovery time, or for 7.5 years, with no traces found in the liver or other organs [56]. Carrageenan degradation is usually realized by gamma irradiation, acid hydrolysis, enzymatic hydrolysis with carrageenase and commercially available enzymes, or oxidative degradation, and is influenced by the carrageenans’ conformation, concentration, molecular weight, viscosity, and stability of the contained glycosidic linkages [48,59]. Furthermore, the biological properties of carrageenan could be modified as a result of the degradation process. Depolymerized CGs have anti-HIV effects that are influenced by their molecular weight, and the carrageenans’ oligomers could expedite the wound healing process [48]. Even though it has anti-HIV activity, because it has anticoagulant properties, it is not recommended as a therapeutic drug for AIDS due to the side effects [56]. On the other hand, because CGs have antiviral activity against HIV and human papilloma virus, and they have a low cytotoxicity level, they might be used as microbiocidal condom coatings and vaginal lubricant gels [48,60].

Because CG seems like a promising pharmaceutical excipient, various studies and several reviews have been published to date [1,37,39,40,46,47,48,50,55,56,57,59,61,62,63,64,65], but fewer than those regarding alginate, HPMC, or chitosan. Their biological activities are determined by the sulfate content. Anticoagulant, immunomodulatory, antitumor, antithrombotic, antioxidant, antiviral, cytoprotective, and growth factor activities were reported for CGs [1,39,48,56,62]. The inhibitory effect might be due to the anionic CG molecules that interact with the positively charged virus or surface of a cell. Even though CG cannot always prevent viruses from attaching or entering the host cell, it inhibits viral protein synthesis inside the cell [56]. Commercial CG oligosaccharides can inhibit tumor growth through immune system regulation instead of directly killing the tumor cell [66].Carrageenans offer antimicrobial effects against some pathogenic bacteria, such as *Aeromonas hydrophila*, enterotoxigenic *E. coli*, *Salmonella enteritidis*, *S. typhimurium*, *Staphylococcus aureus*, and *Vibrio mimicus*. Significant bacterial growth inhibition was realized especially under I-CG influence, and a bacteriostatic effect was observed using CGs against *S. enteritidis*, an important role being played by the sulfate residues inside the CG [45].

The blending of polyethylene oxide (POLYOX™) with carrageenan (CG), sodium alginate (SA), chitosan (CS), and hydroxypropylmethyl-cellulose (HPMC) was used to develop hydrogel films with the addition of glycerol (GLY) in order to plasticize the formulations. The compounds obtained showed impressive swelling ability, good control of drug release over 72 h, and satisfactory flexibility, transparency, and bioadhesiveness. These films were loaded either with diclofenac for the inflammatory phase in wound healing to relieve pain and reduce the local swelling, or with streptomycin to target specific bacterial infections. With regard to drug diffusion, the results were impressive, over a significant period of time, with a crucial impact on inflammation and bacterial infection. Consequently, film removal for drug supplementation was no longer needed [64,65]. The proper swelling that rose with time is an indispensable characteristic for efficient mucoadhesion and extended drug release. POLYOX-CG films were tested at a 7.3 pH in PBS (phosphate buffered saline), with a higher swelling (hydration) ability for the plasticized ones, mostly because glycerol leads to higher flexibility that increases the spaces inside the film so a higher water uptake is possible. This mechanism of water intake ratio increasing with time is extremely useful for highly exuding wounds. This film displayed a sleek, uniform surface morphology, great transparency and elasticity, elegant appearance, and sufficient mechanical (tensile) characteristics that displayed the wanted properties of an ideal dressing. The aforementioned great transparency allows better observation of the healing process. The significant mucoadhesive force and wound exudate absorption capacity protects the wounds from many external factors that might harm the injured area. The drugs had an antibacterial inhibition effect against *S. aureus*, *P. aeruginosa*, and *E.coli*, the causative microorganisms most found in chronic wounds [46,67].

Carrageenan seems to be the best polymer for twice-a-day doses of metoprolol tartrate delivery in three-layered matrix tablets, with controlled drug release and improved linearity, mainly because of the CG super case II release mechanism and its better match with the target release profile. Metoprolol tartrate is used in angina pectoris, arrhythmia, and hypertension treatment. Complexes containing metoprolol showed a gelation behavior after hydration. During a compared study, formulations with carrageenan showed a slower metoprolol drug release in a hydrochloric acid buffer media with pH 1.2 compared with many polymers, including pectin and gums [68].

### 3.1. Carrageenan Nanoformulations

#### 3.1.1. Nanofibers

The unique characteristics possessed by carrageenans due to exhibiting antiviral, immunomodulatory, anticoagulant, antioxidant, and anticancer properties have prompted the attempt of developing carrageenan-based polymeric nanofiber structures [69]. The underlying reason is that polymerization of various biomaterials with a wide array of biological qualities into nanofibers has been successfully implemented in a number of cases, including polycaprolactone (PCL), poly-lactic-co-glycolic acid (PLGA), poly-L-lactic acid (PLLA), silk fibroin, collagen, chitosan, alginate, hyaluronic acid (HA), and cellulose [70], resulting in a diversity of potential applications.

Carrageenan-based nanofibers are obtained in a similar fashion to other polysaccharide-based nanofibers, by electrospinning [71]. This technique originates from the observations regarding the extraction of fine fibers from viscoelastic fluids in the presence of an external electric field. Extensive research has led to a much more precise definition of the underlying processes, and the resulting materials have been implemented in a wide series of applications, including the development of wound dressings. The principle behind electrospinning revolves around the effects of a strong electrical field on a droplet of viscoelastic liquid. In most setups, the droplet is formed at the tip of a blunt needle connected to a syringe holding the solution employed. The droplet is electrified due to the generated field, which results in the elongation of its initial pendant shape. This is mediated by the repulsion of the same sign charges accumulated on the droplet surface as a result of its exposure to the electrical field and to the interaction of the forces resulting from the surface tension inherent to the used viscoelastic liquid.

**Figure 4:**
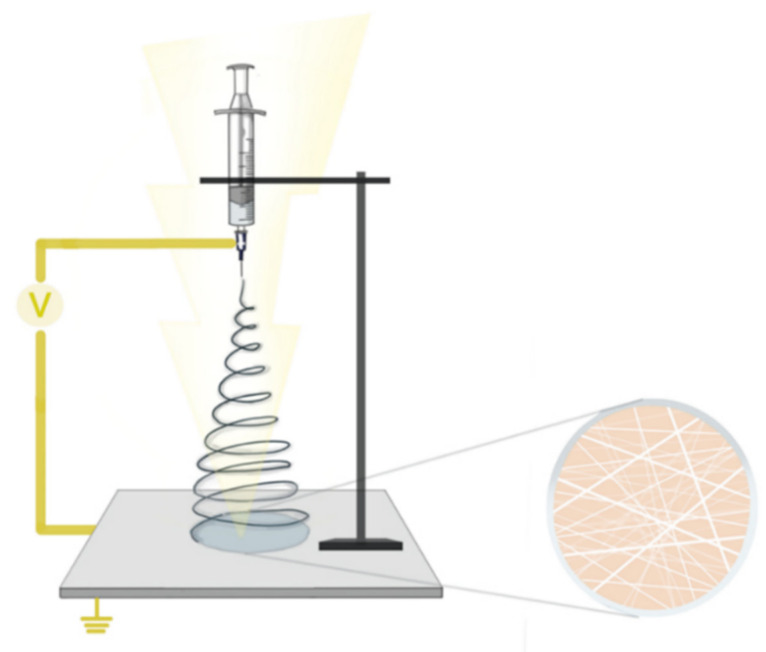
Carrageenan nanofibers electrospinning principle.

As a result of the polymer solution or melt droplet’s transformation from a spherical to a conical shape when subjected to a high electric field, this elongation results in the production of a Taylor cone. The Taylor cone will eject the constituting liquid inside in a very fine jet that emerges from the tip of the cone if the electrical field intensity is raised past a critical point. [72,73,74,75]. The resulting jet is stretched and thinned under the continuous effect of the electrical field, but stays unfragmented due to the viscous and elastic properties of the solution. When polymer solutions are jetted, the solvents rapidly evaporate, leaving thin nanofibers made up of the polymer used. The resulted nanofibers are then deposited on a collector plate situated at a variable distance from the tip of the syringe needle [76]. This process is illustrated in Figure 4.

Native k-carrageenan is the most frequently used variant of CGs in electrospinning applications. Commercially available food-grade kappa- and lambda-CGs have failed to produce nanofibers by electrospinning, possibly due to their highly hydrophilic nature and their thin-shearing behavior at high shear rates [77,78]. In order to improve the capacity of forming electrospun fibers, K-CG has been blended with various biopolymers in different proportions [79,80,81,82,83,84].

K-CG can be combined with polyvinyl alcohol (PVA), frequently used as a co-spinning material in conjunction with several polymers to improve the produced nanofibers via electrospinning [77]. When used in a proportion of 70:30 (PVA:CG), Salihfudin et al. studied the effect of different solvents and PVA types on the production of nanofibers resulting from this particular crosslink ratio, and concluded that nanofibers produced with a blend between fully hydrolyzed PVA (PVA-FH) and CG produced uniform and smooth nanofiber at a 12 *w*/*v*% PVA-FH concentration [79].

Madruga et al. presented a method of producing nanofibers based on a blend of PVA and carboxymethyl-kappa-carrageenan (CMKC) without generating hazardous waste. The ratios used were 1:0, 1:0.25, 1:0.4, 1:0.5, and 1:0.75 (*w*/*w* PVA:CMKC) in aqueous solution. They generated a 15 kV electrical field in the electrospinning process, set the tip-to-collector distance to 15.0 cm, and pumped the solution at a flow rate of 1 mL/h, thus obtaining nanofibers with a diameter below 300 nm. The resulting nanofibers were adequate for use in contact with biological systems, and the incorporation of CMKC in PVA nanofibers proved to have useful implications for the biological acceptance of the material. Furthermore, PVA:CMKC blends exhibited a series of effects on cellular metabolism and behavior, including response to differentiation signals, postulating the idea of a possible application in tissue engineering using stem cells [81].

Another technique that enables the use of carrageenan in electrospinning involves oxidation by exposure to sodium periodate (NaIO_4_). Abou-Okeil et al. used a blend of PVA, oxidized K-CG (OKC), and hyaluronic acid (HA) in various proportions, which they pumped at 0.5 mL/h while subjected to a 17.5 kV electrical field with a tip-to-collector distance of 10 cm. The uniform nanofibers generated in the case of a 2.5:2.5:5 ratio of HA:OKC:PVA were proven to act as an antibacterial against *S. aureus* (Gram positive bacteria) and *E. coli* (Gram negative bacteria), opening a new perspective of various biomedical uses [77].

Furthermore, the properties of CG-based nanofibers can be enhanced by doping, i.e., the addition of substances with specific pharmacological properties. Through this process, the resulting biomaterials acquire the involved pharmacological properties. A study conducted by Gouda et al. experimented with the addition of reduced graphene oxide (prGO) to a PVA—iota-carrageenan (I-CG) nanofiber scaffold [80]. The nanofibers obtained by Gouda et al., named MNS, proved to have efficient wound dressing capabilities with enhanced bioactivity due to the addition of prGO [80]. Graphene-based materials possess a well-documented beneficial effect on wound healing, skin repair, and infection prophylaxis when used in wound dressings [85,86,87]. Doping has also been used in instances where the observed effect was the slow release of particular pharmacologically active agents, such as topical application, where the development of novel hydrogels has shown great potential [88].

From the aspects presented thus far, it becomes discernible that several parameters can be fine-tuned when generating electrospun carrageenan nanofibers. The most apparent concern is the precise composition of the polymer solution, with regard to polymer concentration, employed solvent, and secondary ingredients. Moreover, the voltage, the distance between the syringe tip and the collector plate, and technical and environmental factors can all have an impact on the quality of the obtained nanofibers [75].

Table 2 contains a series of experiments in the aforementioned regards as described in the literature and the corresponding biological effects correlated with the studied parameters.

#### 3.1.2. Nanoparticles

The addition of certain nanoparticles in nanomaterials has different complementary or modulating effects on the properties of the resulting compounds. The use of nanoparticles in order to enhance the properties of wound dressing materials is a subject of significant current interest [93,94]. Some experimental nanoparticles integrated into carrageenan-based nanoformulations are presented in Table 3, along with the properties associated with each nanoparticle.

Researchers discovered that using nanoparticles in a hydrogel nanocomposite form of CGs (CGs by themselves; with CG plus polyvinyl alcohol and ferrous oxide nanoparticles; with CG and chitosan; with CG and polyacrylic acid; or with CG and calcium carbonate) showed excellent results in pharmaceutical and biomedical fields such as cell, drug, and protein delivery, Gram-positive *S. aureus* inactivation, tissue regeneration, and cancer and AIDS therapy [50].

Zinc oxide and copper oxide nanoparticles were blended into functional hydrogels and dry films based on carrageenan. If KCl was added as a crosslinker, the gel strength increased, resulting in translucent (if the nanoparticles were present) or transparent (if not) hydrogels; if the hydrogel films were dry, they would exhibit a free-standing and flexible aspect. The antibacterial activity of the nanoparticle-loaded hydrogels was observed against *E. coli* and *Listeria monocytogenes*. The hydrogel films loaded with zinc oxide nanoparticles displayed higher antimicrobial, mechanical strength, thermal stability, UV screening, and water holding characteristics than the ones loaded with copper oxide nanoparticles. The swelling ratio of carrageenan films depends on their composition, as follows: without KCl, the swelling ratio was 2665%; after KCl was added, it increased to 2980%; and after zinc oxide nanoparticles were added, the swelling ratio reached 3535%. These films or hydrogels might be used in food packaging, cosmetic, or biomedical industries [117].

In addition to all the aforementioned types of CG blends for wound healing, researchers also focused on other biological aspects of using this biopolymer. Some of them, such as biocompatibility, immunogenicity, and local properties, are presented in the following section.

K-carrageenan hydrogels using poly (*N*-vinyl-2-pyrrolidone), potassium chloride, and polyethylene glycol that have been prepared for wound healing applications showed great results during the in vitro tests, such as effective fluid absorption, high elasticity, flexibility, soft texture, good mechanical strength, good transparency, and adhesion to the wound surface with a pain-free removal. When this hydrogel is able to pass the biocompatibility, bacterial infection prevention, water evaporation control, and gas permeability tests in both in vitro and in vivo studies, then this wound dressing will have a higher chance of approval. The first results showed that this CG form meets some of the most important ideal dressing properties such as good transparency and mechanical strength, pain-free removal, effective fluid absorbance, high elasticity, and flexibility [61].

The antiviral activity of CG manifests through selective inhibition of several enveloped viruses, such as herpes simplex virus, human rhinoviruses, human cytomegalovirus, human immunodeficiency virus, and papilloma virus (in vitro), mainly through hindering the bond or entry of virions inside the cell [56].

Tablets with L-carrageenan and 63% diltiazem hydrochloride had very fast disintegration and drug release rates in both 1.2 and 6.8 pH media, with complete drug release in an hour, and this complex did not form a gel [48]. The mechanisms behind this controlled drug release are thought to be the slow erosion or dissolution of the tablet surface complexes, the water uptake, and interactions between all the involved molecules that might lead to a 24-h release time, while the ions contained in the medium influenced the separation of drug–polymer complexes [48].

Apart from their multiple uses, there are some concerns about the use of CGs, mainly because the biocompatibility is questioned due to the inflammatory reactions observed in several studies and the toxicological characteristics that are very different between the low molecular mass degraded CG and the food-grade ones [1]; however, this toxicity is very low, without being teratogenic [56]. Degraded CG may lead to significant ulceration of the colon mucosa and might cause serious health issues [48]. Some of the adverse reactions included necrosis of human colonic epithelial cells, either cell lines or primary cells, and it seems that intestinal ulcerations and tumors are related to degraded and undegraded CGs exposure [46]. Gelatin/K-CG sponges give an acute inflammation response (not chronic), angiogenesis, and fibroblast migration [63]. The main biological activities that carrageenans are involved in are summarized in Table 4.

In addition to wound healing applications, K-carrageenan presents remarkable results in the field of regenerative medicine [124]. Thus, K-CG has been reported to have osteogenic potential because it induced the metabolic activity of pre-osteoblast cells [125]. Silk fibroin/K-CG electrospun nanofibers showed great osteogenic properties [126]. Furthermore, the use of a “bioactive composite scaffold with enhanced biomimetic mineralization” that also had silk fibroin “led to a bone-like apatite layer” deposit after 7 days [127]. Jafari et al. [69] mention certain in vitro studies that used: methacrylate-K-CG hydrogel for soft tissue engineering, bioprinting, and suture-less adhesives; CG/Whitlockite nanoparticles hydrogel for bone tissue engineering and drug delivery with a great expression of both early and late osteogenic markers; and a K-CG hydrogel for cartilage regeneration that displayed very low cytotoxicity [69]. CG also had a beneficial effect on chondrogenic and osteogenic induction in a mesenchymal stem cell culture study [128]. Crosslinked nanofibers with PCL/I-CG, PHB/K-CG, and PHBV/K-CG were also used for bone tissue engineering [89,90].

By using a CG-alginate hydrogel to deliver cells, Popa et al. [129] created a new matrix with tissue/cartilage engineering potential. Popa et al. [130] demonstrated, using in vitro and in vivo studies that a K-CG hydrogel is biocompatible and could be used in tissue engineering [130,131]. One promising approach is the use of K-CG as bioink for 3D bioprinting, with excellent results regarding the uniform spread of cells on the scaffold, biocompatibility, and cell attachment and viability [132].

## 4. Conclusions

Carrageenans are high molecular weight polymers extracted from several species of red algae. Their applications were studied intensively because most types are able to form gels and have a hydrophilic character. In addition to using carrageenans in the food industry, they have also been studied for their pharmacological applications. The biomedical field uses these biopolymers as conventional and advanced drug delivery systems, and as key ingredients for creating hydrogels, scaffolds, matrixes, and nanoformulations for tissue repair, such as healing wounds or bone and cartilage regeneration. Their biological activities are determined by the sulfate content, and many studies are focused on the anticoagulant, anti-inflammatory, anti-pathogenic, antitumor, antithrombotic, antioxidant, antiviral, cytoprotective, and immunomodulatory effects carrageenans have. Other compounds, polymers, or materials have been incorporated into different types and formulations of carrageenans, and the synergistic effects enhanced the quality of the results.

Even though carrageenans have a low cytotoxic level against healthy cells, further studies will most likely create a blend that is 100% biocompatible. These biopolymers’ versatility in types, formulations, and applications appears to make them ideal candidates as prime ingredients for development or improvement of a novel wound healing biomaterial. We are eager to see the new ideas that will arise in order to address all the questionable aspects of using them in wound healing, and to soon see carrageenan-based wound healing medical devices available over-the-counter worldwide.

## Figures and Tables

**Table 1:** General characteristics of carrageenans.

Characteristics	Description	Reference
Type	Sulfated polysaccharides	[45,48]
Source	Red algae (*Chondrus, Eucheuma*, *Furcellaria*, *Gigartina*, *Hypnea*)	[40,45,48,49]
Structure	Alternating 3-linked b-D-galactopyranose (G-units) and 4-linked a-D-galactopyranose (D-units) or 4-linked 3,6-anhydro-a-D-galactopyranose (DA-units)	[39,41,45,50]
Molecular weight	High and variable, A CG 416.39 g/mol B CG 336.33 g/mol I CG 946.74 g/mol K CG 788.64 g/mol L CG 594.52 g/mol	[43,44,51,52,53,54]
Functional groups	3,6-anhydrogalactose, ester sulfate, 3,6-anhydrogalactose-2-sulfate, and galactose-4-sulfate	[39,44,49,55]
Solubility	Water-soluble, insoluble in organic solvents, oil or fats K-, I-CG—dissolves in Na^+^; L, T, X—insoluble	[45]
Gelation factors	Influenced by temperature, concentration, ions (Na^+^, K^+^, and Ca^2+^), and pH Thermo-reversible. L, T, X—do not form a gel M, N, K, I—gel with K^+^ or Ca^2+^ ions, or alkali treatment K-CG in K^+^ and Na^=^ salts presence—weak gel K-, I-CG in K^+^ and Ca^2+^ ions—higher gelling temperature K-CG 35 to 65 °C I-CG: higher Viscosities: 5–800 cps in 1.5% solutions at 75 °C	[44,46,48,56]
Swelling factors	Levels of hydrophilic sulfate groups, salt form, Fe_3_O_4_ nanoparticles, molecular weight	[39,42,46,57]
Dissolution factors/time	Types/number of solutes present, temperature, carrageenan type, molecular weight, concentration; slow	[46,49,53,56]

**Table 2:** Biological effects of various types of carrageenans.

CG Used	Technique	Studied Effects	Ref
OKC	K-CG oxidation—exposure to NaIO_4_ (1:0.5 ratio; at 40 °C; for 3 h; at pH 3), crosslinking with PVA and HA (HA:OKC:PVA—0:4:6; 1:3:6; 2:2:6; 3:1:6; 2.5:2.5:5; 3.5:3.5:3). Electrospinning: 5 mL syringe; 22G needle; 0.5 mL/h at 25 ± 2 °C; 10 cm tip-to-collector; 17.5 kV DC voltage	Antibacterial properties	[77]
K-CG	Solvents: distilled H_2_O, with and without sonication; NaCl solution; crosslinking with fully or partially hydrolyzed PVA (PVA:K-CG 70:30)	Drug and nutrient delivery ability	[79]
CMKC	Crosslinked with PVA; PVA:CMKC—1:0; 1:0.25; 1:0.4; 1:0.5; 1:0.75. Electrospinning: 19G needle; 1 mL/h at 20 ± 2 °C; 15 cm tip-to-collector 15 kV DC voltage	Cytocompatibility, biodegradability, cell growth, cell adhesion, adipose-derived stem cells’ response to osteogenic differentiation signals	[81]
I-CG	Crosslinked with PVA and addition of graphene oxide (PVA:I-CG:GO—95:3:2). Electrospinning: 15 cm tip-to-collector 20 kV DC voltage	Wound healing, skin repair, antimicrobial properties	[80]
I-CG	Crosslinking with polycaprolactone (PCL); PCL:I-CG—100:0; 95:5; 90:10; 85:15; 80:20; 0:100. Electrospinning: 10; 15; 18 cm tip-to-collector 20 kV DC voltage	Biocompatibility, bone tissue growth	[89]
K-CG	Crosslinked with polyhydroxybutyrate (PHB) or polyhydroxybutyrate valerate (PHBV); PHB:K-CG and PHBV:K-CG—100:0; 90:10; 80:20; 70:30. Electrospinning: 1 mLSyringe dispensed at 3.5 mL/h (PHB/K-CG) and 3.0 mL/h (PHBV/K-CG); 15 cm tip-to-collector; 20 kV DC voltage	Bone tissue engineering	[90]
K-CG	Crosslinked with PHB, caffeic acid (CA), and quaternized chitosan (QCh). Electrospinning: 2 mL/h; 15 cm tip-to-collector; 25 kV DC voltage	Antimicrobial and antioxidant properties	[91]
K-CG	Crosslinked with polydioxanone (PDX); PDX:K-CG—100:0; 90:10; 80:20; 70:30. Electrospinning: 6 mL/h; 15 cm tip-to-collector; 25 kV DC voltage	Viability and differentiation of SaOS-2 preosteoblasts	[92]

**Table 3:** Properties of carrageenan nanoparticle compositions.

Nanoparticles Used	Application	Studied Properties	Ref
Chitosan capped sulfur particles and grapefruit seed extract	Hydrogel films Animal study	Wound healing effect, mechanical strength, increased swelling ratio and ultraviolet barrier properties, decreased water vapor permeability and water solubility	[95]
Polydopamine modified ZnO nanoparticles	Sprayable bioadhesive hydrogel	Mechanical, antibacterial, and cellular properties, blood clotting ability, and biocompatibility	[96]
L-glutamic acid	Sprayable bioadhesive hydrogel	Wound healing	[96]
2D-nanosilicates	Injectable hydrogels for cellular delivery for cartilage tissue regeneration and 3D bioprinting	Shear-thinning characteristics, enhanced mechanical stiffness, elastomeric properties, and physiological stability	[97]
2D-nanosilicates	Injectable hydrogels for hemostasis and tissue regeneration	Mechanical properties, stiffness, protein adsorption, cell adhesion and spreading, increased platelet binding and reduced blood clotting time. Suppression of entrapping vascular endothelial growth factor (VEGF), in vitro tissue regeneration, and wound healing.	[98]
2D-nanosilicates	Bone-cartilage interface tissue engineering	Shear-thinning characteristics, increased the mechanical stiffness, mechanical properties, microstructures, cell adhesion characteristics	[99]
Dopamine functionalized graphene oxide	Injectable hydrogels	Compressive strength and toughness, enhanced in vitro fibroblast proliferation and spreading	[100]
Halloysite nanotubes	Nanocomposite film for tissue engineering	Biocompatibility, mechanical properties, cellular functions	[101,102]
Whitlockite nanoparticles	Injectable hydrogels	Mechanical stability, biocompatibility, protein adsorption, stimulation of osteogenesis and angiogenesis	[103]
Hydroxyapatite nanoparticles	Sustained release drug delivery hydrogels	Sustained release of hydrogel-loaded ciprofloxacin as opposed to burst-release of other hydrogels	[104]
Gold particles	Injectable hydrogels	Electrical conductance, cell growth, and attachment	[105]
Gold particles	Drug delivery hydrogels	Drug release kinetics (diclofenac sodium)	[106]
Magnetic nanofillers (Fe_3_O_4_ nanoparticles)	Drug delivery hydrogels	Drug release kinetics (methylene blue, diclofenac sodium)	[107,108]
MgO nanoparticles	Drug delivery hydrogels	Drug release kinetics (methylene blue)	[109]
Super paramagnetic iron oxide nanoparticles	Drug delivery hydrogels	Stimulus-dependent (magnetic field, temperature, and pH-sensitive) drug release, biocompatibility	[110]
CaCO_3_ -based nanoporous microparticles	Cancer cell targeting drug delivery nanocomposites	Drug release and cell targeting capabilities (doxorubicin)	[111]
Maghemite	Drug delivery nanocomposites	Drug delivery in cancer therapy, biocompatibility	[112]
Selenium	Nanocomposite for tissue engineering	Biochemical properties, osteoblast cell growth	[113,114]
Silver particles	Hydrogel beads	Antibacterial activity, biological safety	[115]
Silver particles	Wound dressing	Antimicrobial effectiveness and physical properties	[116]
ZnO, CuO	Hydrogel and dry films	Mechanical, UV-screening, water-holding, thermal stability, and antimicrobial properties	[117]
Silver particles and divalent cations (MgCl_2_, CuCl_2_, CaCl_2_)	Wound dressing material (hydrogels)	Biocompatibility, tissue regeneration	[118]
Cellulose nanocrystals and silver nanoparticles	Wound dressing material (hydrogels)	Mechanical characteristics, nanocomposite drug release, antimicrobial properties	[119]

**Table 4:** Biological characteristics of carrageenans.

Properties	Description	Reference
Formulations	3D scaffolds, beads, drug-loaded plasticized films, fibers, gels, hydrogels, nanofibers, nanoparticles, PVP-KCG, three-layered matrix, and sponges	[48,56,61,68]
Topical biocompatibility	Low toxicity but non-teratogenic, may cause inflammation and adverse effects on human intestinal epithelial cells	[48,61]
Local properties	Drug delivery, anticoagulant, anti-HIV, antioxidant, antithrombotic, antitumor, and antiviral effect	[1,39,46,48,56,62,120]
Mechanisms	Super case II release mechanism; hydrolysis of glycosidic bonds at pH ≤ 3.0; desulfation by sulfatases; anionic CG molecules interact with the positively charged virus or cell surface	[40,56,68,120]
Immunogenicity	Interfere with antigens lowering the normal immune function Interact with the virus cell surface or its positive charges and prevent host cells from being penetrated by the virus	[56,121]
Anti-infectious properties	Bacteriostatic: *Salmonella enteritidis*; antimicrobial: *Aeromonas hydrophila*, *e*nterotoxigenic *E. coli*, *Listeria monocytogenes, Salmonella enteritidis*, *S. typhimurium*, *S. aureus*, *P. aeruginosa*, *Vibrio mimicus*	[45,117]
Anti-inflammatory properties	Induce inflammation (paw edema), but interfere with NSAIDs. If loaded with diclofenac, they reduce inflammation.	[48,56,122,123]
3D scaffolds	Alginate–carrageenan mix, gelatin/K-CG sponges, K-CG/calcium phosphate, hydrogel beads and fibers, metoprolol tartrate delivery in 3-layered matrix tablets, PVP-KCG	[1,56,63,68,117]
Elimination	Fecal elimination after oral intake	[56]
